# A Tale of Two City Blocks: Differences in Immature and Adult Mosquito Abundances between Socioeconomically Different Urban Blocks in Baltimore (Maryland, USA)

**DOI:** 10.3390/ijerph110303256

**Published:** 2014-03-19

**Authors:** Brian Becker, Paul T. Leisnham, Shannon L. LaDeau

**Affiliations:** 1 Cary Institute of Ecosystem Studies, 2801 Sharon Turnpike, Millbrook, NY 12545, USA; E-Mail: brianbecker2000@gmail.com; 2 Department of Environmental Science and Technology, University of Maryland, College Park, MD 20742, USA; E-Mail: LEISNHAM@umd.edu

**Keywords:** mosquito, vector, urban decay, *Aedes albopictus*, *Culex pipiens*

## Abstract

Infrastructure degradation in many post-industrial cities has increased the availability of potential mosquito habitats, including container habitats that support infestations of invasive disease-vectors. This study is unique in examining both immature and adult mosquito abundance across the fine-scale variability in socio-economic condition that occurs block-to-block in many cities. We hypothesized that abundant garbage associated with infrastructure degradation would support greater mosquito production but instead, found more mosquito larvae and host-seeking adults (86%) in parcels across the higher socio-economic, low-decay block. *Aedes albopictus* and *Culex pipiens* were 5.61 (*p* < 0.001) and 4.60 (*p* = 0.001) times more abundant, respectively. Most discarded (garbage) containers were dry during peak mosquito production, which occurred during the 5th hottest July on record. Containers associated with human residence were more likely to hold water and contain immature mosquitoes. We propose that mosquito production switches from rain-fed unmanaged containers early in the season to container habitats that are purposefully shaded or watered by mid-season. This study suggests that residents living in higher socioeconomic areas with low urban decay may be at greater risk of mosquito-borne disease during peak mosquito production when local container habitats are effectively decoupled from environmental constraints.

## 1. Introduction

More than 80% of the population of the United States lives in cities [1]. However, over the past several decades eleven of the fifteen largest cities in the United States have experienced population declines [2] and similar urban declines have occurred globally [3]. These declines have been associated with the disappearance of industrial jobs [4], government policies and spending programs that subsidize out-migration to the suburbs [5], and a history of real estate practices that have resulted in racial segregation and neighborhood decline [6,7,8]. During population shrinkage, cities typically experience infrastructure abandonment and associated decay that can significantly alter the biotic environment [9]. This process of urban decay tends to exacerbate itself, causing increasing inequities in social, economic, and environmental conditions [4,10,11]. The most visible sign of this process is often the deterioration of buildings and associated accumulation of garbage. Vacant buildings thus not only serve as outcomes of urban decline, but as markers of the symptom [12,13]. The abandonment of one building can adversely affect nearby residents, often leading to further abandonments and a number of other issues, including increased crime and exposure to disease vectors [14,15,16]. 

The emergence and re-emergence of mosquito-borne disease has increasingly been associated with urban landscapes, including West Nile virus (WNV), dengue virus, La Crosse virus (LACV), and chikungunya virus [17,18,19,20,21,22]. While host composition is important for zoonotic pathogen amplification [23,24,25,26], the production of mosquito vector species in close proximity to humans is a fundamental determinant of the distribution and incidence of human disease [27,28,29].

The Asian tiger mosquito, *Aedes albopictus* (Skuse) and northern house mosquito *Culex pipiens* L., readily occupy human dominated landscapes. Both species utilize artificial water-filled containers (*i.e*., trash, bird baths, planters) during immature development e.g., [16,30], and as adults, take bloodmeals from humans, domestic pets, and urban wildlife [27]. *Aedes albopictus* was first documented in the continental United States in Texas in 1985, and has since spread throughout cities across the south-eastern and mid-Atlantic United States to become the predominant human-biting urban mosquito [16,31,32,33,34]. *Aedes albopictus* is a competent vector for pathogens found in the eastern U.S., including WNV, LACV, and Eastern equine encephalitis [17,35,36], as well as for potential emergent pathogens such as chikungunya and dengue viruses [29,37]. *Aedes albopictus* commonly co-occur with the resident invasive mosquito *Culex pipiens,* which invaded North America over 200 years ago and is common in cities throughout the northern United States [38]. Laboratory and field studies implicate *Cx. pipiens* as the principal WNV vector in this region [35,39,40,41].

**Figure 1:**
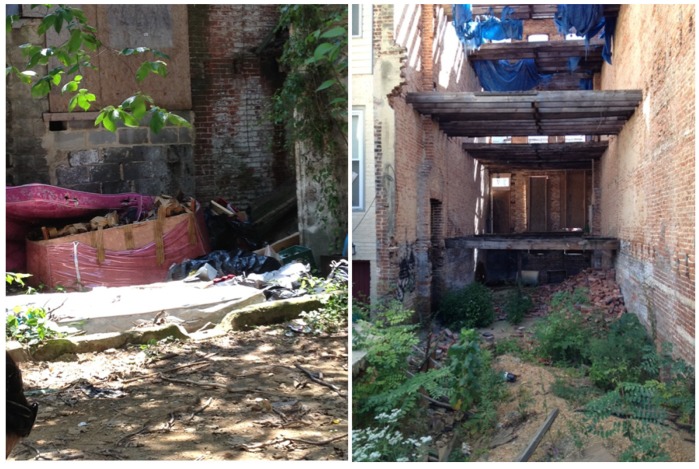
Degraded infrastructure in Baltimore, MD. Source: S. LaDeau.

Vegetation, abundance of water-filled containers, and proximity to standing water have all been associated with the distribution and abundance of vector species [30,33,42,43]. Adult mosquitoesrequire access to vegetation for food and resting sites, aquatic habitats for developmental stages (eggs and larvae), and host blood meals. However, ecological processes occurring at early developmental life-stages likely play a primary role in regulating the distribution and abundance of adult mosquitoes [44,45]. The rate of immature development and pupation depends on abiotic conditions (e.g., water quality, temperature), biotic processes (e.g., microbial food resources, competition, predation) and their interactions [45,46,47,48,49,50,51,52,53,54]. Habitat that supports both immature development and adult mosquito populations is likely heterogeneous across an urban landscape but remains understudied [55,56]. Vegetation cover, container habitat, and densities of both predators and host species can vary between neighborhoods [16,33,57]. The quality and abundance of individual container habitats or adult resting sites vary at even finer spatial scales [30,58,59]. Previous studies have found both positive and negative associations among suitable mosquito habitat, mosquito abundance, and neighborhood socio-economic status [14,16,30,59,60,61,62,63,64]. However, few studies have gone beyond income to examine the specific associations between the physical symptoms of urban poverty and the abundance of blood-seeking adult mosquitoes [61]. Symptoms of physical disrepair such as holes, water damage, peeling paint, and garbage accumulation have been associated with the presence and abundance of indoor pests [65,66]. However, the specific importance of infrastructure deterioration for maintaining mosquito infestations is not well understood. Vacant buildings are increasingly abundant in many U.S. cities and when abandoned, the associated physical disrepair may indicate increased mosquito habitat (e.g., Figure 1). Degraded structures are often associated with unmanaged and semi-permanent garbage collections that can hold water, facilitate immature mosquito development [16] and increased production of biting adults. 

This study examines the relationship between the physical degradation of an urban residential landscape and the production and composition of mosquito communities at both larval and adult stages. Specifically, we compared immature and adult mosquito abundance on individual parcels across two city blocks in Baltimore, Maryland that varied in urban decay, as defined by the density of roofless, vacant houses. We expected that the buildings and associated garbage would reflect increased habitat for immature mosquito development. We hypothesized that neighborhoods with more roofless, vacant buildings would have more unmanaged larval habitat and greater relative abundance of adult mosquitoes than neighborhoods with fewer vacant houses. 

## 2. Experimental Section

**Figure 2:**
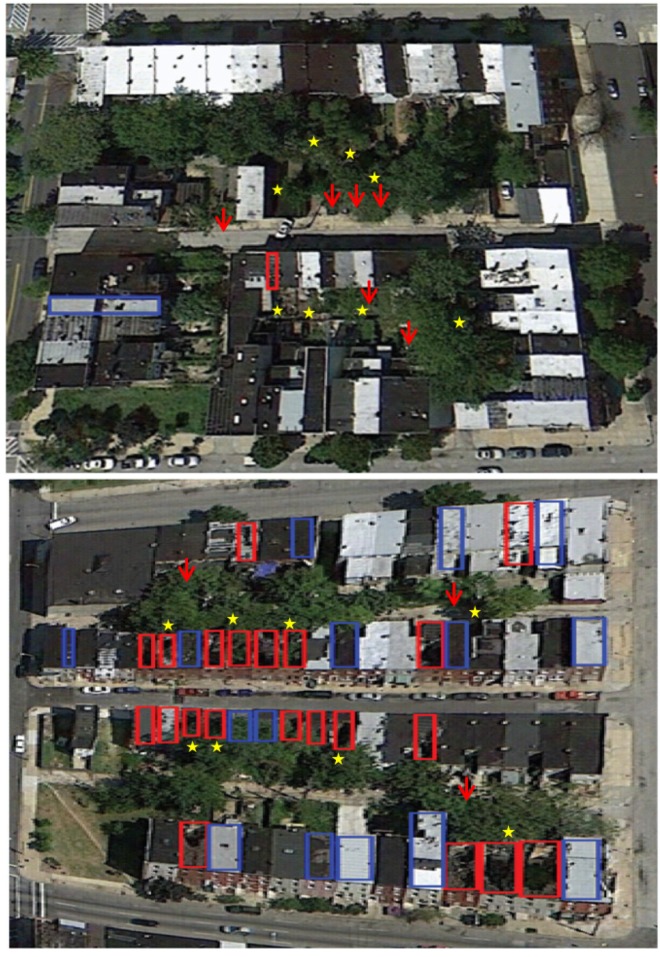
Aerial photos *circa* 2010 of the study blocks (Google Earth) with low (top) and high (bottom) urban decay symptoms. Red and blue boxes indicate vacant buildings with damaged or intact roofs, respectively. Stars represent adult trap sites and red arrows indicate mosquito positive larval habitat.

Baltimore City, Maryland lost 31.4% of its population between 1950 and 2000 and has a 19.5% household poverty rate, which is well above the national average (11.1%; US Census Bureau, 2010). The city has more than 16,000 vacant and abandoned buildings [67], which are an easily identifiable metric of urban infrastructure decay in impoverished neighborhoods. We focused on two city blocks located in West Baltimore that were similar in area and original row house structure. We quantified the total number of vacant buildings on each block as a proxy measure of urban decay using information provided by the City of Baltimore’s Open Data Catalog [68]. We used Google Earth (images acquired in 2010) to identity roof damage (Figure 2) and then confirmed counts of vacant and deteriorating buildings during a site visit in June 2012. We selected focal blocks that contained relatively high and low numbers of vacant and deteriorating buildings (Table 1). As part of a larger study, Knowledge, Attitude, and Practice (KAP) surveys were conducted with one resident at each participating household (unpublished data). Voluntary information on education and income levels confirm that higher socio-economic households were generally located on the low decay block (52% college degree and 35% earning more than 95,000 annually *versus* 0 households reporting these levels in the high decay block). We also ensured visually that blocks adjacent to each of our focal blocks were similar in number of vacant buildings to the adjacent focal block. The study blocks were located approximately 800 meters apart to maximize spatial independence at the scale of mosquito dispersal but maintain continuity in physiographic characteristics*.* The predominant mosquito species from previous larval surveys in West Baltimore [16] was *Ae. albopictus,* which usually disperses no more than 150–200 m from larval development sites and rarely more than 800 m [69,70,71]. 

**Table 1:** Block-level summaries.

Block Characteristic	Low-decay	High-decay
Area (hectares)	1.31	1.28
Parcels (includes empty lots)	58	77
Total Buildings	54	70
Vacant Buildings	2	36
Vacant Buildings with missing roof	1	21

### 2.1. Adult Mosquito Samples

Mosquitoes were trapped at two locations per block for two days during the week starting 22 July 2012 and two days during the following week starting, 29 July 2012. Adult trap locations were selected to minimize trap exposure to sunlight, wind, and rain, since traps set in exposed areas may collect fewer mosquitoes [72]. Due to this relatively low sample size, we limit interpretation of our results to the comparison between high and low urban decay sites during this specific and important time period, which has been previously demonstrated to be the peak season for both *Ae. albopictus* and *Cx. pipiens* in this region [73,74,75]. 

Traps were set at 2 PM and collected the next day at 9 AM. To maximize our ability to collect both *Culex* and *Aedes* mosquitoes, traps were set in pairs consisting of a CDC Light Trap and a Biogents ^®^ (BG) Sentinel trap located within 0.5 meters of each other and baited with CO_2_ (300 g dry ice in a blue insulated canister). Replicate trap pairs were placed at two trap locations per block on each trap day, for a total of 8 samples per trap type per block. Collected samples were labeled and transported on dry ice back to a lab where they were identified and enumerated. Mean daily rainfall during July 2012 was 28 mm, which is consistent with the 5-year July mean [76]. However, there was only one precipitation event (5.84 cm) during the two week sampling period of this study, which occurred on 26 July, after both the immature survey (see below) and the initial adult collection. Local mean temperature in July 2012 was 27.44 °C, the 5th hottest July in over 100 years on record [76].

### 2.2. Immature Mosquito Samples

Immature mosquito surveys were conducted at the parcel-level on each of our focal blocks during the first week of adult sampling 22–25 July 2012. Each parcel was surveyed for standing water and all accessible water-holding containers were sampled at: (1) Occupied houses whose residents permitted access, (2) vacant lots with permission from Baltimore City, and (3) public access alleys. Container habitats were recorded as either being useful (e.g., garden planter, trash can, drain spout), discarded garbage (e.g., cup, can, food wrapper, tire) or structural disrepair (building material, puddle). After the water was homogenized, up to 1 L was collected from each container and mosquito larvae and pupae were enumerated and identified to species (larvae) or genus (pupae) level [38]. Nine (of 28, 32.1%) container habitats held more than 1 L of water and the total larvae for these samples was calculated as the density of sampled larvae per L multiplied by the total volume (L) of water. All but two containers held 0.02 L–7.0 L water. Two containers were much larger, including a trash can that was estimated to hold 50 L of water and a bucket with 20 L of water. In calculating the total mosquito abundance of these two containers, we limited the total volume to 10 L because mosquitoes appeared to be at a similar density as our sampled L only in the top 10 L of water. We consider this a reasonable approach that is supported by other studies that demonstrate that mosquito density should decline with depth [77]. 

All analyses were conducted in the R statistical package using standard generalized (Poisson) linear regression (GLM function). The unit of inference was considered to be the individual parcel, and container density and mosquito densities (total and by species) were summed on a per parcel basis. In each model, a binary variable was used to assess differences between the high (0) and low (1) decay block.

## 3. Results and Discussion

### 3.1. Adult Sampling

A total of 1,162 adult mosquitoes were collected, of which 588 were female *Ae. albopictus* (50.6%) and 549 male *Ae. albopictus* (47.2%). The second most common species collected was *Cx. pipiens,* with only 29 females sampled (0 males). Other species collected were *Aedes vexans* (<0.1%)*, Ochlerotatus cantator* (<0.1%)*,* and *Ochlerotatus sollicitans* (<0.1%). The majority of all samples were collected in BG Sentinel traps (97% and 98% of *Ae. albopictus* and *Cx. pipiens*, respectively). Further analyses use summed mosquito abundance from each trap pair for each sampling date.

Eighty-six percent of the adult mosquitoes were collected from the low-decay block. Female *Ae. albopictus* were 5.61 times more abundant (z = −5.875, *p* < 0.001) and *Cx. pipiens* were 4.60 times more abundant (z = −3.191, *p* = 0.001) in the low-decay block *vs.* the high-decay block. The proportion of male *Ae. albopictus* relative to females in the same trap ranged from 0.07 to 0.71 (mean proportion male 0.49 ± 0.21). Mean male-to-female ratio was not statistically different between samples from the high- *vs.* low-decay blocks (0.46 ± 0.16 *vs.* 0.37 ± 0.23, respectively). No males of other species were collected.

### 3.2. Immature Sampling

A total of 1,275 fourth-instar larvae and 79 pupae were sampled from container habitats across both blocks (772 larvae and 65 pupae in the low-decay block, 503 larvae and 14 pupae in the high-decay block). Of these, 379 larvae (29.7%) and 45 (56.9%) pupae were *Ae. albopictus,* and 832 larvae (65.3%) and 34 (43.0%) pupae were *Cx. pipiens*. Other species collected included fourth instar *Culex restuans* (0.04%), *Aedes triseriatus* (<0.01%) and unidentified mosquito species (0.01%). 

Yard access varied between the two blocks, with greater numbers of fenced yards and fewer residents home during the day to grant access in the low-decay block *vs.* the high-decay block. We sampled ten parcels (17.24%) in the low-decay block, including eight with occupied buildings and two grass lots. Water-holding containers were found on all parcels sampled on the low-decay block (Table 2). With the exception of one discarded tire, all mosquito infested containers in the low-decay block were classified as useful and appeared to be used for yard maintenance and care (e.g., trash cans, bird baths, buckets, planters). While there was more abundant potential container habitat in discarded garbage and structural disrepair around the vacant buildings in the high-decay block (personal observation), most of these containers were dry when surveyed. Of the 42 parcels surveyed in the high-decay block, 16.7% had water-holding containers and only 7.1% had immature mosquitoes. 

**Table 2:** Larval sampling metrics.

Block Metric	Low-decay	High-decay
Parcels surveyed (#)	17.24% (10)	58.33% (42)
Parcels with accessible water , % surveyed	100.00%	16.67%
Parcels with mosquito larvae, % surveyed	60.00%	7.14%
Containers per parcel, mean (se)	2.57 (0.26)	0.26 (0.08)
Containers with larvae, %	50.00%	22.22%
Containers with pupae, %	27.78%	22.22%
Mean larvae per surveyed parcel, *Ae. albopictus* (se)	14.78 (6.36)	2.69 (1.92)
Mean larvae per surveyed parcel, *Cx. pipiens* (se)	26.22 (14.12)	8.57 (6.30)
Mean adult per trap night, Ae. albopictus (se)	70.57 (13.45)	11.00 (3.40)
Mean adult per trap night, *Cx. pipiens* (se)	3.29 (1.32)	0.63 (0.50)

Mosquito pupae were only found in seven containers sampled on two parcels in each neighborhood; six of the pupae positive containers were categorized as useful (uncovered trash cans and one plastic tarp) and one was a large puddle in an alley in the high-decay block. All were adjacent to or on occupied parcels. Total numbers of *Aedes* and *Culex* pupae were similar (45 *versus* 48), although *Aedes* pupae were only sampled from container habitat in the low-decay block. *Aedes* and *Culex* pupae were not sampled concurrently from any container, although earlier immature stages (larvae) were commonly found together. 

The relative abundance of both *Ae. albopictus* (z = 13.80, *p* < 0.0001) and *Cx. pipiens* (z = 13.78, *p* < 0.0001) larvae per parcel was significantly higher in the low-decay block compared to the high-decay block. However, the presence of immatures in a particular container was not well predicted by the block decay status (*Ae. albopictus: p* = 0.081 and *Cx. pipiens p* = 0.080, respectively). Larvae of both species co-occurred in most (73%) mosquito-positive containers. However, abundance of *Ae. albopictus* larvae was negatively associated with container volume (z = 4.098, *p* = 0.00004) while abundance of *Cx. pipiens* larvae was positively related to container volume (z = 10.354, *p* < 0.0001). Both species occurred at higher abundances in useful *vs.* discarded garbage containers during this survey (*Aedes*: z = 4.053, *p* = 0.00005; *Culex* z = −2.606, 0.0092).

### 3.3. Discussion

Relative abundances of immature and adult mosquitoes were consistent between blocks, with greater abundance of both in the low-decay *vs.* high-decay block. However, interspecific adult abundance was not well predicted by the immature sampling. Although *Ae. albopictus* comprised almost all the collected adults, *Cx. pipiens* was quite abundant at the larval stage. However, *Culex* pupae were sampled from parcels on both blocks, while *Aedes* pupae were not collected from any containers in the high-decay block. The apparent mismatch between immature and adult presence and relative abundances could be a result of interspecific differences in immature development rates, survival, trap sensitivity or adult dispersal. While our CO_2_ baited CDC traps were intended to target *Culex* samples, we actually sampled more of both species in the BGS traps. Future efforts will need to employ gravid traps to directly sample ovipositing females, in addition to BGS traps. Exactly how abiotic and biotic processes act on the immature stages of mosquitoes to regulate the abundances of biting adults is often complex and our findings highlight a clear research need for evaluating these processes in an urban landscape. 

The simplest ecological mechanism driving adult abundance is immature survival, whereby low immature survival directly leads to low adult emergence. Our findings suggest greater *Ae. albopictus* survival to emergence and that adult mosquito production in the high-decay block is limited by persistence of aquatic habitat during an exceptionally hot and dry period. While garbage containers were abundant, they were almost always dry (personal observation). In general, disused garbage containers are likely to dry more quickly than larger containers, making them less important mosquito habitat in dry conditions [33,78]. In addition to affecting adult abundance through immature survival, abiotic and biotic processes acting on the immature stages of mosquitoes can indirectly affect adult abundances through changes in adult body size, longevity and fecundity [46,79,80], as well as affect adult biting rates and vector competence [81,82,83,84]. Evaluating the effects of container mosquito ecology on adult life-history and disease transmission is particularly important within urban landscapes where mosquitoes are in close proximity to humans and habitat gradients vary with social context.

Our findings regarding container habitat importance were similar to other surveys in the region that identify planters, buckets and uncovered trash cans as important *Ae. albopictus* habitat [30,33], although somewhat in contrast to findings that discarded garbage and infrastructure decay may also create productive habitat [16,30,62]. Garden planters and buckets often held a higher volume of water than what precipitation could have provided. If planters and other container habitats in the low-decay block are regularly watered or purposefully shaded by residents, then this common mosquito habitat can be effectively decoupled from the dry weather conditions that limited mosquito production in disused garbage containers. 

While heat and low precipitation levels are normal for July, 2012 was hotter than average [76]. Higher temperatures can increase immature development rate [85,86], although heat can also directly reduce persistence of aquatic container habitat [85]. Numerous studies have shown that numbers of water-filled containers and larval mosquitoes are both less abundant in dry conditions [73,87,88]. Thus, extreme heat may reduce adult mosquito production in urban areas where disused containers are the predominant habitat for immature development. While the abundant container habitat in the high-decay block was dry during our study, it may play an important role in early season population growth and in seasons with cooler, wetter conditions as indicated in prior studies [16]. 

This study is among the few to compare both immature and adult mosquito abundances across fine-scale variation in socio-economic context. By being conducted during dry conditions it removes large variations in mosquito abundances that may be seen between periods of dry and wet conditions over an entire season, which in turn can mask differences across fine spatial scales such as city blocks. By examining both immature and adult abundances, this study highlights some important aspects of mosquito ecology in socio-economically diverse urban landscapes. Populations are likely to be regulated at multiple life-stages in a species-specific manner and resident-based yard upkeep and gardening may be important for maintaining mosquito populations during hot and dry periods. 

## 4. Conclusions

Vacant and abandoned buildings are a visible sign of neighborhood socioeconomic status in many post-industrial cities. Prior studies in this region of the U.S. have reported positive association between neighborhood socio-economic status and mosquito infestation [16,33,89]. We hypothesized that the infrastructure damage and garbage accumulation associated with vacant buildings would provide more unmanaged container habitat that would result in higher immature densities and greater production of adult mosquitoes. While vacant houses in the high-decay (lower socio-economic status) block in this study did have a higher proportion of containers categorized as garbage relative to the low-decay block, we found both more total water-holding containers per parcel and greater densities of immature mosquitoes per container in the higher socio-economic status (low-decay) block. Containers associated with human residence and outdoor activity (e.g., planters, trash cans) were more likely to both hold water and contain immature mosquitoes at the time of our study. 

The vast majority of immature and all adult mosquitoes were either the invasive *Ae. albopictus* or resident invasive *Cx. pipiens.* Both of these species utilize water-holding artificial containers in urban landscapes and are known vectors for a range of arboviruses, including the endemic West Nile virus [35]. Our data suggest that residents on the low-decay block may be at greater immediate risk of mosquito-borne disease than residents in the (lower socio-economic status) high-decay block. 

Our results may not reflect differences in mosquito abundances under wet conditions; previous work in this region suggests that lower socioeconomic neighborhoods can support greater larval habitat over the entire mosquito season [16]. We’ve highlighted a critical need for developing better understanding of what spatial and temporal scales are important for forecasting and managing mosquito populations and disease risk in urban landscapes. Future studies should rigorously explore spatial dynamics of both immature and adult mosquito abundances during both dry and wet periods, preferably over multiple summer seasons, to gain a fuller understanding of temporal and spatial mosquitoes populations in urban landscapes. 
